# The Mechanisms and Management of Age-Related Oxidative Stress in Male Hypogonadism Associated with Non-communicable Chronic Disease

**DOI:** 10.3390/antiox10111834

**Published:** 2021-11-18

**Authors:** Kristian Leisegang, Shubhadeep Roychoudhury, Petr Slama, Renata Finelli

**Affiliations:** 1 School of Natural Medicine, Faculty of Community and Health Sciences, Bellville, Cape Town 7535, South Africa; 2 Department of Life Science and Bioinformatics, Assam University, Silchar 788011, India; shubhadeep1@gmail.com; 3 Department of Animal Morphology, Physiology and Genetics, Faculty of AgriSciences, Mendel University in Brno, 61300 Brno, Czech Republic; 4 Gamma Analisi Cliniche S.r.l., 81100 Caserta, Italy; finelli.renata@gmail.com

**Keywords:** antioxidants, noncommunicable chronic disease, nutrition, phytonutrients, testosterone, testosterone replacement therapy

## Abstract

Androgens have diverse functions in muscle physiology, lean body mass, the regulation of adipose tissue, bone density, neurocognitive regulation, and spermatogenesis, the male reproductive and sexual function. Male hypogonadism, characterized by reduced testosterone, is commonly seen in ageing males, and has a complex relationship as a risk factor and a comorbidity in age-related noncommunicable chronic diseases (NCDs), such as obesity, metabolic syndrome, type 2 diabetes, and malignancy. Oxidative stress, as a significant contributor to the ageing process, is a common feature between ageing and NCDs, and the related comorbidities, including hypertension, dyslipidemia, hyperglycemia, hyperinsulinemia, and chronic inflammation. Oxidative stress may also be a mediator of hypogonadism in males. Consequently, the management of oxidative stress may represent a novel therapeutic approach in this context. Therefore, this narrative review aims to discuss the mechanisms of age-related oxidative stress in male hypogonadism associated with NCDs and discusses current and potential approaches for the clinical management of these patients, which may include conventional hormone replacement therapy, nutrition and lifestyle changes, adherence to the optimal body mass index, and dietary antioxidant supplementation and/or phytomedicines.

## 1. Introduction

Steroid sex hormones are classified as androgens, estrogens, and progestogens. Although all three classes are important in male and female physiology, androgens are associated with "musculisation" effects and are considered primarily male sex hormones [1]. Androgens have diverse functions in muscle physiology, lean body mass, the regulation of adipose tissue, bone density, neurocognitive regulation, and spermatogenesis, male reproductive and sexual function [2].

When testosterone synthesis is impaired, a condition of hypogonadism arises that affects quality of life and wellbeing [3]. Because of the importance of testosterone in male physiology, hypogonadism further leads to increased fat accumulation, a reduction in lean body mass, and osteoporosis. Hypogonadism may also arise as a consequence of the ageing process, which can be described as the gradual deterioration in biological function over time, reducing quality of life and increasing the risk of degenerative noncommunicable chronic diseases (NCDs) [4]. In fact, ageing has an important relationship as both a risk factor and/or a comorbidity with NCDs, including obesity, metabolic syndrome, type-2 diabetes mellitus (T2DM), and numerous malignancies [2,4].

All these conditions have in common an imbalance in the redox homeostasis in favor of higher levels of oxidants, leading to the development of oxidative stress [5]. Oxidative stress is a leading mechanism that drives the ageing process, with a complex relationship in the pathogenesis of age-related NCDs. Moreover, it may have an important role in age-related hypogonadism associated with the increased risk of NCDs [6,7,8]. Hence, in this context, the management of oxidative stress may represent a novel approach for the treatment of patients. Therefore, this narrative review aims to discuss the mechanisms of age-related oxidative stress in male hypogonadism associated with NCDs and to discuss current and potential novel management approaches that may include conventional hormone replacement therapy, nutrition and lifestyle changes, weight management, and dietary antioxidant supplementation and/or phytomedicines.

## 2. Steroidogenesis and Male Hypogonadism

**Figure 1:**
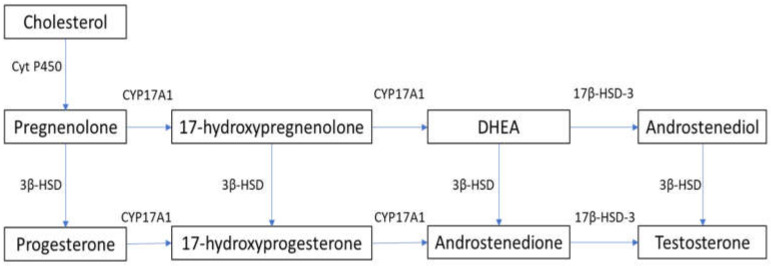
Steroidogenesis pathway. 3β-HSD: 3β-hydroxysteroid dehydrogenase; 17β-HSD-3: 17β-hydroxysteroid dehydrogenase-3; Cyt P450: cytochrome P450; CYP17A1: 17α-hydroxylase; DHEA: dehydroepiandrosterone.

In males, testosterone is synthesized primarily in Leydig cells through LH receptor (LHR) binding, with the activation of G-coupled protein and adenylyl cyclase, which increases intracellular cyclic adenosine monophosphate (cAMP). This leads to the activation of the steroidogenic acute regulatory (StAR) protein that mediates the mitochondrial uptake of cholesterol, which is converted to pregnenolone by the cytochrome P450 side chain cleavage enzyme [9,10]. In humans, 17α-hydroxylase (CYP17A1) converts pregnenolone to 17α-hydroxy-pregnenolone and androgen dehydroepiandrosterone (DHEA). DHEA is converted to androstenedione via 3β-hydroxysteroid dehydrogenase (3β-HSD), and then to testosterone via 17β-hydroxysteroid dehydrogenase-3 (17β-HSD-3) [9,10]. The steroidogenesis pathway is summarized in Figure 1. cAMP activation is required for the expression of steroidogenesis enzymes [11]. Androgens exist both in free form and bound to serum proteins. Although approximately 98% of testosterone is bound to albumin or sex-hormone-binding globulin (SHBG), about 2% of circulating testosterone is not bound to serum proteins and is able to penetrate into cells and exert its metabolic effects [12].

Male hypogonadism is a clinical syndrome caused by a disruption of the hypothalamic–pituitary–gonadal (HPG) axis that affects the testicular synthesis of testosterone [3]. Various terminologies are used to describe this syndrome, including testicular failure, androgen deficiency syndrome, testosterone deficiency syndrome, andropause, androgen deficiency in ageing males (ADAM), and late-onset hypogonadism (LOH) [3]. Hypogonadism is estimated to affect up to 12% of male adults in the general population, and the incidence is expected to increase in the future [3], mainly because of the rise in the population aged 65 years and over.

The classification of hypogonadism is based on testicular or non-testicular causes. Testicular failure is classified as primary (hypergonadotropic) hypogonadism, and the causes include Klinefelter syndrome, Sertoli-cell-only syndrome, cryptorchidism, testicular trauma, mumps orchitis, radiation or chemotherapy treatment, and autoimmune diseases [3]. Pituitary or hypothalamic causes are classified as secondary (hypogonadotropic) hypogonadism, and include Kalman’s syndrome, pituitary adenoma, hyperprolactinaemia, and medications [3]. Although often reported as secondary hypogonadism, the nontesticular causes of "mixed" hypogonadism can be caused by ageing, excessive alcohol consumption, and corticosteroid treatment [3]. Important clinical associations with hypogonadism as risk factors and/or comorbidities include obesity, metabolic syndrome, T2DM, cardiovascular disease, and osteoporosis [3]. Furthermore, lower levels of testosterone in healthy men are a predictor of obesity, metabolic syndrome, and related comorbidities [13]. Hypogonadism is also associated with environmental exposures that induce oxidative stress, which can result in male infertility. This includes exposure to air pollution, pesticides, heavy metals, radiation, and, particularly, endocrine-disrupting chemicals [14].

Hypogonadism presents clinically with sexual dysfunction, prominently including erectile dysfunction, infertility, increased adiposity with decreased muscle mass, reduced bone density, and osteoporosis, fatigue, and depression [3]. Diagnosis is confirmed with a reduced serum total testosterone on two separate occasions [15], while the determination of the serum LH differentiates primary (increased LH: hypergonadotropic) from secondary (reduced LH: hypogonadotropic) hypogonadism [15]. Age-associated hypogonadism may be characterized by normal or low-normal levels of LH [15].

## 3. Oxidative Stress and Hypogonadism

**Figure 2:**
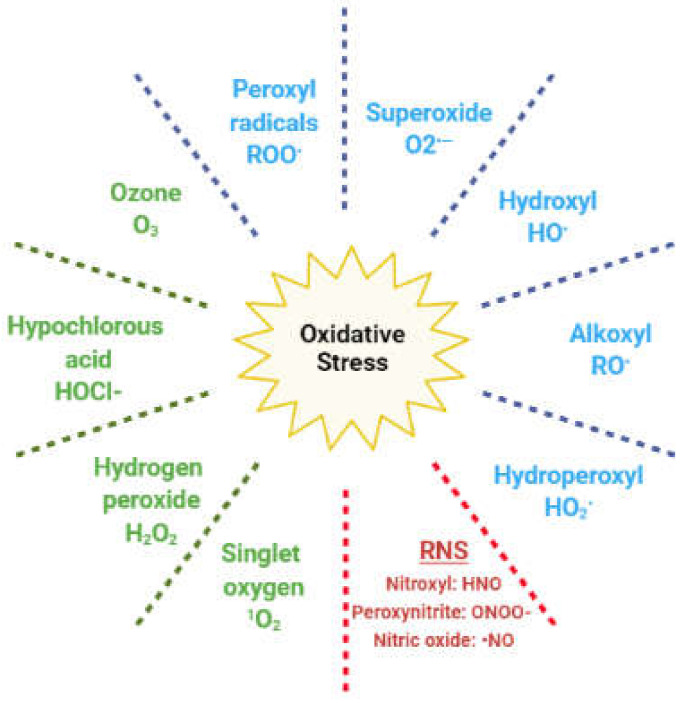
Reactive species responsible for oxidative stress. In blue: radical oxygen species; in green: nonradical oxygen species; in red: reactive nitrogen species (RNS).

In living cells, redox (reduction and oxidation) reactions mediate numerous physiological pathways; hence, the intracellular levels of oxidants and antioxidants play an important role in this fine regulation [5,16]. Reactive oxygen species (ROS) are oxygen-based oxidants that are generated during cellular metabolism, predominantly in the mitochondria. They act as physiological mediators in several processes, such as immune regulation, inflammation, apoptosis, and the regulation of genetic expression, among others [5,16]. The ROS family includes both radical and nonradical species (Figure 2).

The former are molecules with unpaired electrons in the outer orbit; hence, they easily react with any other cellular molecule, including lipids, proteins, and DNA. Nonradical species include hydrogen peroxide (H_2_O_2_), which can react with ferrous ions in the Fenton reaction and lead to the generation of hydroxyl radical. Other oxidants originate from nitrogen and are classified as reactive nitrogen species (RNS) [5]. Redox homeostasis is maintained by enzymatic and nonenzymatic antioxidant compounds, which are endogenously generated, or introduced exogenously through the diet (Table 1) [17].

When the fine redox equilibrium is shifted in favour of oxidants through increased ROS or reduced antioxidants, a condition of oxidative stress arises. As numerous cellular signaling pathways respond to variations in redox status, oxidative stress can consequently result in the disruption of the cellular signalling and cause cellular damage [18]. Several studies have described the ROS-dependent regulation of molecular pathways, such as extracellular signal-regulated kinases (ERK), c-Jun N-terminal kinases (JNK), phosphatidylinositol 3-Kinase (PI3K)/Akt, p38, p53, protein-kinase C, phospholipase C, nuclear factor kB (NF kB), and JAK/STAT. These can have either a pro- or anti-apoptotic effect (for review on the topic, see [19,20]. In clinical practice, oxidative stress has been widely described as a contributing factor in several pathological conditions, such as cardiovascular, autoimmune, and neurodegenerative disorders, as well as cancer, diabetes, and infertility [21,22,23,24,25].

ROS are physiologically produced during the enzymatic reactions of steroidogenesis. Monooxygenase reactions require electron donation from NADPH through adrenodoxin reductase and adrenodoxin [26,27]. Here, electron leakage results in the generation of superoxide and other ROS [26,28]. Experiments in a mouse Leydig tumor (MA-10) cell line demonstrated that ROS mediate the cAMP-activation of RAS and the phosphorylation of ERK1/2, after the binding of the LH hormone on Leydig cell receptors [29]. The activation of these pathways is reported to positively modulate the proliferation and survival of Leydig cells, as well as steroidogenesis [30,31].

**Figure 3:**
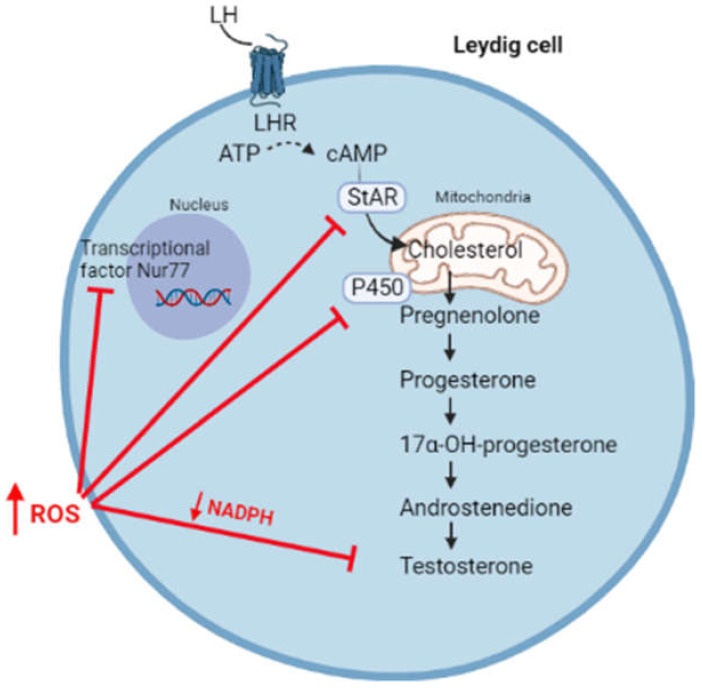
High levels of ROS affect steroidogenesis through the inhibition of transcriptional factor Nur77, the synthesis of StAR, enzymatic P450 activity, and by reducing the levels of NADPH cofactor.

However, a switch of the redox status towards oxidative stress can affect steroidogenesis, resulting in the reduced synthesis of androgens (Figure 3).

High levels of ROS hyperactivates the JNK signalling, which suppresses the activity of Nur77, a transcriptional factor regulating the expression of several steroidogenic enzymes [32]. Moreover, free radicals can oxidize the heme catalytic group of cytochrome P450, resulting in enzymatic inactivation [33,34]. By using oxidative agents, Chen et al. reported an increased phosphorylation of the MAPK family members (ERK1/2, JNK, and p38) associated with reduced testosterone production in MA-10 cells, highlighting the importance of the redox status in the regulation of steroidogenesis [35]. In this regard, it has been suggested that the activation of the p38 pathway is responsible for reduced testosterone production, possibly through the activation of cyclo-oxygenase2 (COX2) [35,36,37]. In fact, the overexpression of COX-2 has been associated with the reduced expression of StAR and, consequently, with reduced testosterone synthesis [38,39]. Moreover, high levels of ROS are responsible for the depolarization of the mitochondrial membrane, associated with the post-transcriptional inhibition of the StAR protein [40].

ROS scavenging relies on antioxidant systems. The reduced synthesis of testosterone was observed in Nrf2 knock-out mice, where Nrf2 is a transcription factor regulating the expression of antioxidant systems in response to oxidative stress [41]. Experiments conducted in young and aged rats showed that the depletion of glutathione (GSH) was accompanied by reduced testosterone synthesis [42]. In addition, the switch towards an oxidative status with reduced levels of NADPH can indirectly affect steroidogenesis through the inhibition of enzymatic activities. In fact, NADPH is an important cofactor of several endogenous antioxidants, such as GSH and thioredoxin, as well as enzymes involved in steroidogenesis [10,43].

## 4. The Complex Association between Ageing, Hypogonadism, Non-communicable Chronic Diseases, and Oxidative Stress

Ageing is a complex, multifactorial, and gradual deterioration in biological function over time, which affects the neurological, immune, and endocrine systems, and is responsible for cellular dysfunction and genetic mutations [8,44]. This results in a time-dependent reduction in quality of life and an increased risk of degenerative NCDs, prominently including obesity, metabolic syndrome, T2DM, cardiovascular disease, osteoporosis, osteoarthritis, numerous malignancies, and neurodegeneration [6,7,8]. Although there is no accepted single theory of ageing to fully explain the accumulation of damaged cellular structures, oxidative stress has been reported to be closely related to increased cellular senescence and age-related degenerative diseases [6,7,8]. In fact, oxidative stress correlates with an increasing body mass index (BMI), and serum glucose in animals and humans, and also mediates the metabolic syndrome phenotype [45]. Numerous single nucleotide polymorphisms (SNPs) involved in the regulation of antioxidant defences are associated with an increased risk for obesity, systemic inflammation, and insulin resistance [46]. Mitochondrial dysfunction and the accumulation of mitochondrial DNA damage are considered important in the ageing process and NCDs [44]. Hyperglycaemia further increases oxidative stress through the glycation of proteins and the subsequent oxidative metabolism [22].

Obesity, metabolic syndrome, T2DM, and numerous malignancies have common risk factors. These include an increased consumption of energy-dense foods, a sedentary lifestyle, alcohol and tobacco use, exposure to environmental toxins and endocrine-disrupting chemicals, as well as numerous medications [47,48]. These lifestyle and environmental factors are also known to induce oxidative stress and inflammation [45].

Although frequently caused by overnutrition, obesity and T2DM paradoxically present with micronutrient deficiencies [49]. Prominent deficient micronutrients include vitamins A, B, C, and D, selenium, zinc, and chromium [49]. Deficiencies in these important exogenous antioxidants contribute to the reduced activity of tyrosine kinase, impaired insulin signalling, β-cell dysfunction, muscle catabolism, and increased intracellular calcium, contributing to the development of metabolic syndrome and T2DM [50].

The role of oxidative stress in T2DM has been described in the literature [51]. In fact, patients reportedly showed mitochondrial dysfunction in leukocytes because of the lower mitochondrial membrane potential, associated with higher ROS generation and a lower expression of the antioxidant systems [52,53]. In DM, high levels of glucose are converted to sorbitol, consequently reducing the NADPH and GSH levels [54]. ROS also inhibit the enzymatic action of glucose-6-phosphate dehydrogenase, the main enzyme involved in the pentose phosphate pathway and the synthesis of NADPH [55]. This significantly impairs the reductive cellular potential. Similarly, high levels of ROS alter physiological pathways, leading to the increased activation of protein kinase C. This has been identified as one of the main molecular mediators of diabetic vascular complications [54]. Furthermore, advanced glycation end products (AGE) generated in the T2DM condition are highly reactive molecules that enhance the generation of ROS [56].

In human observational studies, oxidative stress in obesity and T2DM correlates with male hypogonadism [57,58]. Similarly, ageing in males is associated with reduced levels of androgens (late-onset hypogonadism), particularly testosterone and DHEA, and increases in LH, FSH, and SHBG. As dihydrotestosterone (DHT) levels remain relatively constant, there is a decline in the testosterone/DHT ratio [4,59]. Late-onset hypogonadism is a common presentation, affecting up to 25% of elderly males, with more than 10% presenting with the clinical signs of hypogonadism [9]. The reasons why ageing results in hypogonadism are multiple. First, the hypothalamic secretion of GnRH may be dysregulated in elderly men, reducing the frequency of LH pulses [60]. In addition, elderly men show higher levels of SHBG, which reduces the concentration of free testosterone [61]. Furthermore, the synthesis of testosterone by Leydig cells is significantly affected by ageing. Animal studies show that steroidogenic synthesis is impaired by the reduced synthesis of cAMP, StAR protein, and other translocator proteins binding to cholesterol, as well as mitochondrial enzymes [62]. In addition, autophagy (a process where unneeded cellular components are degraded and recycled) is reportedly decreased in aged animals and in vitro models [63,64], resulting in reduced cholesterol uptake and utilization, and testosterone synthesis [65].

In this context, oxidative stress plays an important role. Studies conducted in rats showed that the depletion of the GSH antioxidant was associated with reduced testosterone synthesis, which was reverted once that antioxidant pool was restored [42]. Oxidative stress disrupts mitochondrial function and mitochondrial membrane potential, ATP synthesis, and the mitochondrial calcium concentrations in Leydig cells. This leads to reduced steroidogenesis through interference with StAR protein transcription, subsequent cAMP production, and the activity of cytochrome P450 [40,66,67,68,69]. The androgen axis is also reportedly involved in the maintenance of cellular DNA integrity, as it increases the transcription of those genes responsible for repairing single- and double- DNA strand breaks [70]. As a consequence, reduced testosterone synthesis may affect DNA integrity and result in increased DNA fragmentation and mutation rates. Hydrogen peroxide, which can be generated by testicular macrophages, affects the enzymatic activity of 3β-hydroxysteroid dehydrogenase, superoxide dismutase, catalase and glutathione-S-transferase, and increases lipid peroxidation and apoptosis, with negative repercussions on steroidogenesis [68,71]. A detailed understanding of these pathways may open the possibility for novel treatments to improve steroidogenesis in ageing males, as there is the potential to target these pathways to modulate the consequences of ageing and improve reproductive potential and sexual function.

Male reproduction can be affected in cases of obesity and metabolic syndrome. In fact, the adipose tissue present in excess expresses the enzyme aromatase, which mediates the conversion of testosterone to estradiol. Moreover, hyperinsulinemia is associated with reduced SHBG levels [72]. This results in the suppression of the reproductive hormonal axis and, consequently, hypogonadism. In addition, conditions such as obesity and metabolic syndrome are usually associated with inadequate antioxidant intake through the diet [73]. Conversely, oxidative stress and inflammation can be triggered by the intake of excessive macronutrients (overnutrition), such as high-fat diets and/or high-carbohydrate diets, with increased NF-kβ activity [74,75]. The high levels of leptin, hyperglycemia, and endothelial dysfunction contribute to the impairment of the antioxidant enzymatic activity, the increased ROS generation, and the establishment of an inflammatory and oxidative microenvironment, with repercussions for male fertility [76]. Lower levels of testosterone have been associated with the rise in insulin resistance, hyperglycemia, and T2DM [52,77]. In T2DM, a proinflammatory condition is also established, with the expression of inflammatory markers (such as TNFα and c reactive protein). A negative correlation between such markers and total testosterone was reported [78,79], with their concentration being reduced by testosterone treatment in T2DM patients [80].

Oxidative stress, along with chronic inflammation, has been widely reported to be associated with cancer [81]. Oxidative stress can initiate malignant transformation and cellular proliferation, but can also induce cellular death [82]. The ROS-mediated DNA damage includes the generation of modified oxidized DNA bases, whose levels have been reported to be higher in several types of cancer [81]. Furthermore, ROS act as molecular mediators in physiological process, such as apoptosis, proliferation, and angiogenesis. Hence, high ROS levels significantly increase the rate of mutations and enhance oncogenic transformation, as well as contribute to chemo- and radio-resistance [81]. Advanced male cancer patients commonly present with hypogonadism, which is associated with reduced muscle strength, overall performance, and quality of life, as well as cancer-related fatigue [83]. This is mainly due to the chronic inflammation associated with malignancy and its treatment [84].

Oxidative stress is therefore a common mediator between physiological ageing, hypogonadism, and the development of NCDs in males. Furthermore, hypogonadism, which is also partly mediated by oxidative stress, is a common consequence of ageing, as well as a contributor to, and/or a consequence of, NCDs in males. In fact, a reduced intake of exogenous antioxidants, or the presence of SNP in endogenous antioxidant genes that promote oxidative stress, are associated with an increased risk of developing obesity, systemic inflammation, insulin resistance, and hyperglycemia, all of which further increase oxidative stress. Therefore, oxidative stress may be an important mediator in the inter-relationships between ageing, hypogonadism, and NCDs that can be a target for management and prevention.

## 5. Management of Male Hypogonadism Associated with Non-communicable Chronic Diseases

### 5.1. Testosterone Replacement Therapy

Pharmaceuticals commonly marketed for hypogonadism include testosterone cypionate (cypionate), testosterone enanthate (enanthate), or testosterone undecanoate as injections, or, alternatively, transdermal administration [15]. In the USA and Europe, only about 10% of males with hypogonadism are being treated with testosterone [85]. In hypogonadal males, testosterone replacement therapy (TRT) is beneficial for weight loss and protection against the loss of lean body mass and bone mass, and is considered more effective than diet alone or bariatric surgery, particularly over the long term [86,87]. However, this improvement is not maintained after the cessation of the TRT [86]. Improvements in metabolic syndrome features and T2DM are also apparent with TRT, improving waist circumference, hypertension, dyslipidemia, blood glucose, glycosylated hemoglobin, and insulin resistance [88,89]. However, the treatment of diabetic males with TRT showed inconsistent results, and routine TRT in diabetic males without clinical symptoms is not currently recommended [90].

The impact of TRT on oxidative stress is unclear. TRT in male patients with postsurgical hypopituitaric hypogonadism results in increased coenzyme Q10 and reduced ROS levels [91]. TM3 Leydig cells exposed to low-dose testosterone concentrations show reduced cellular toxicity through reduced ROS and lipid peroxidation, with increased StAR expression. However, at high doses, the testosterone showed a biphasic response and increased cellular oxidative stress [92]. TRT showed a protective effect against oxidative stress in animal studies. In mice with infertility due to spinal cord injury, TRT improved the testosterone concentration and the markers of oxidative stress [93]. In rats with testicular oxidative stress induced by methotrexate, testosterone protected spermatogenesis via a reduction in testicular inflammation and oxidative stress [94]. Conversely, in healthy adult male rats, testosterone administration increased lipid peroxidation, advanced glycation end products, and the total antioxidant capacity, with the downregulation of StAR and reduced endogenous testosterone production [95]. This may be due to excess testosterone in otherwise healthy animals.

The benefit and long-term safety of TRT in ageing males is unclear [4]. TRT is contraindicated in prostate carcinoma, benign prostate hyperplasia, increased prostate-specific antigens, and a history of cardiovascular events [3,96]. However, at least in the short term, TRT does not appear to promote prostate cancer [97,98]. Importantly, TRT is detrimental to male fertility, and alternative methods to increase testosterone in males who would like to father children are required [99,100]. These may include hCG, GnRH, hMG, and aromatase inhibitors for hypogonadotropic or normogonadotropic hypogonadism, dopamine antagonists for hyperprolactinemia, or the access to assisted reproductive techniques for primary testicular failure [99,100]. In age-related hypogonadism and mild hypogonadism, the long-term benefit-to-risk ratio remains unclear [15]. The use of 5α-reductases, such as finasteride and dutasteride, inhibits testosterone conversion to DHT. This is typically used in cases of benign prostate hyperplasia, lower urinary tract symptoms, and/or androgenetic alopecia management. These drugs result in hepatic fat accumulation and increased glucose synthesis, and a predisposal to insulin resistance with erectile dysfunction [2]. Aromatase inhibitors are also shown to increase testosterone in obese males with hypogonadism [101].

### 5.2. Metformin

Metformin is an effective antiglycemic agent that improves insulin resistance in diabetic and metabolic syndrome patients, and that can lead to weight loss in diabetic and nondiabetic obese patients. Through the activation of AMPK and the inhibition of the NF-κB pathway, metformin improves oxidative stress, inflammation, and the regulation of insulin, glucose, and lipoproteins [102,103]. Particularly, metformin is reported to be more successful in improving the markers of oxidative stress than lifestyle modification [104]. However, its effects on the male reproductive system are poorly understood [105,106,107,108]. On one hand, metformin reportedly improves reproductive function in males with diabetes, including improving the semen parameters, testicular antioxidant function, the intratesticular testosterone concentration, and the serum testosterone concentration [106,108,109]. On the other hand, the activation of AMPK inhibits steroidogenesis through the downregulation of StAR [110,111]. Hence, the impact of metformin on testosterone in males is unclear. Murine and human fetal testicular cells exposed to metformin showed reduced testosterone production [112]. Metformin has also been shown to reduce testosterone levels in vivo, which recovered after the cessation of treatment [112]. In a cohort of middle-aged overweight males with glucose intolerance, metformin was not associated with increased testosterone [113]. Furthermore, metformin may lead to reduced testosterone and libido and erectile function in T2DM [114,115], whereas sulfonylurea, as an alternative, may improve these parameters in T2DM males [115].

### 5.3. Nutrition and Weight Management

Good nutritional habits regulate immune function and redox biology, reducing inflammation and oxidative stress [75]. Mediterranean-based diets are the most studied and recommended, consisting of complex carbohydrates (whole grain), vegetables and fruits, seafood, nuts, seeds, and vegetable oils [75,116,117]. Furthermore, the consumption of antioxidant micronutrients, particularly carotenes, vitamin C and E, and selenium and zinc, with an increased intake of phytochemicals, improves oxidative stress and male reproductive potential [75,116,117,118]. The Mediterranean diet is also associated with a reduced risk for, and the important management of, metabolic syndrome, diabetes, malignancy, and improved longevity [119]. However, high-energy poor-nutrient foods, such as simple sugars, trans fatty acids, and tobacco and alcohol all induce inflammation and oxidative stress [75,118]. Low testosterone in males is predicted with a poor diet, specifically the increased consumption of pastries and breads, dairy, fast foods, and a reduced intake of green vegetables, correlating negatively with lean body mass and positively with BMI, independent of age [13]. Vegan diets may improve SHBG, but appear to have little effect on testosterone [120].

Weight management improves male fertility and endocrine function, where a healthy lifestyle can improve sexual dysfunction and many comorbidities in obesity, metabolic syndrome, and diabetes [121,122]. Furthermore, weight management is important in maintaining normal testosterone and HPG function in obese men, as well as in middle-aged and elderly males [123,124].

As the long-term safety of TRT is unclear in obese males with nonclassical hypogonadism, lifestyle changes or bariatric surgery for weight loss should be the first-line treatments [125]. An appropriate diet, with or without increased physical activity, improves body weight in obese males, with or without metabolic syndrome or T2DM [113,126]. An intensive increase in physical activity may also increase testosterone in obese males [127]. However, bariatric surgery may be more beneficial in some cases [125]. In a meta-analysis, a low-energy diet and bariatric surgery were both found to increase testosterone and SHBG in males undergoing weight loss trials. Bariatric surgery was found to be more effective than low-energy diets, particularly in morbid obesity [128].

Caloric restriction is a significant reduction of the total caloric intake that is done without resulting in micronutrient deficiency, which has been demonstrated to increase lifespans in humans, mammals, and other species [129,130]. Alongside intermittent fasting, caloric restriction reduces inflammation and oxidative stress, and increases endogenous antioxidant levels, most prominently superoxide dismutase. A large number of studies conducted in low-complex models (*C. elegans*, *Drosophila melanogaster*, *S. cerevisiae*), as well as in vertebrates, reported decreased levels of oxidative products and ROS generation in cases of caloric restriction (for a review on the topic, see [131]). This is associated with improvements in obesity and the related metabolic complications [74,132,133]. Caloric restriction may improve male fertility parameters, although this remains unclear. In fact, even if there is a proposed trade-off between the increased longevity associated with caloric restriction and a reduction in fertility, the long-term restriction in Rhesus monkeys showed minimal negative effect on fertility parameters and testosterone over seven years [134]. However, caloric restriction in healthy young men may reduce testosterone [135,136]. In a small study of obese males, caloric restriction increased testosterone and reduced the estrogen concentration, mediated through an improvement in testicular steroidogenesis and a reduction in aromatase activity [137]. However, larger studies are required to confirm this observation. Furthermore, any role of reduced oxidative stress in the improvement of testosterone in obese males undergoing caloric restriction remains unclear and requires further investigation.

Bariatric surgery improves male obesity-associated secondary hypogonadism [138]. This is particularly apparent in morbidly obese males, where hypogonadism is an indication for bariatric surgery, and in over 90% of hypogonadal morbid obese males recovering from hypogonadism [139,140]. This surgery results in reduced BMI and waist circumference, with improved insulin resistance and glucose tolerance, vitamin D, SHBG, FSH and total and free testosterone, inhibin B and AMH, and reduced estrogen and prolactin [138,139,140,141]. Bariatric surgery in obese males also improves sexual dysfunction, including erectile dysfunction, libido, and satisfaction with sexual intercourse [142,143]. Lower levels of seminal lipid peroxidation were reported after bariatric surgery, along with improved semen parameters, mitochondrial activity, and sperm DNA integrity [144], and improved testosterone in male obesity-associated secondary hypogonadal patients [145]. Bariatric surgery further improves the inflammatory and oxidative stress markers in males and females [146,147]. A study conducted in diabetic rats undergoing bariatric surgery described an activation of PPARα, a nuclear transcription factor involved in the oxidative stress response. This resulted in the reduced activation of the NF-kB pathway and, consequently, inflammation, reduced ROS generation, and reduced apoptosis rate [148]. In 2017, a study conducted on 47 men with morbid obesity reported a significant decrease in the salivary oxidative markers (levels of lipid peroxidation, oxidation protein products, and 8-hydroxy-D-guanosine) six months after bariatric surgery [149]. Three months after gastric bypass, the gene expression profiles of obese nondiabetic patients were also reportedly altered, with an increased expression in the genes (SOD2, Sirtuin 1, Sirtuin 3, and Nuclear factor erythroid 2-related factor 2) involved in the cellular response to oxidative stress [150]. Weight loss in bariatric surgery improves the total antioxidant capacity, which correlates with an improvement in the BMI, waist-to-hip circumference, serum insulin, and uric acid [147]. Although bariatric surgery improves inflammatory and oxidative stress markers, an additional supplementation of vitamins may be necessary to maintain this improvement [151]. However, a direct causative relationship between bariatric surgery, a reduction in oxidative stress, and improvements in testosterone has not been established.

Table 2 provides a summary comparison of the impact of TRT, metformin, caloric restriction, and bariatric surgery on obesity, metabolic syndrome, hypogonadism, and oxidative stress. This is a summary based on the current literature available, relevant to the pharmaceutical and nutritional interventions discussed above.

### 5.4. Antioxidants, Micronutrients, and Phytotherapy

Antioxidants may be beneficial in extending the lifespan, and evidence for this includes catechins from green tea, theaflavins from black tea, polyphenols from apples and blueberries, and anthocyanins from black rice, sesemin, curcumin, as well as DHA from marine microalgae [152]. Antioxidants, such as alpha-lipoic acid, arginine, vitamins C and E, carotenoids, coenzyme Q10, lycopene, melatonin, pentoxifylline, resveratrol, selenium, and tocopherols are associated with an improvement in obesity and the metabolic complications and comorbidities [153,154,155,156]. Furthermore, vitamin E has been identified for its ability to maintain β-cell function and improve glucose regulation in diabetics [157].

Antioxidants, and other micronutrients, may be beneficial for reducing testicular oxidative stress and Leydig cell apoptosis. There are few studies investigating the effects of antioxidants on male hypogonadism and the oxidative stress markers in humans. Curcumin nanomicelle improved semen parameters and testosterone levels, reduced FSH, LH, and prolactin, and improved oxidative stress through the reduction of serum MDA, CRP, and TNF and by increasing the serum total antioxidant capacity in infertile males [158]. Animal and *in vitro* studies that have investigated the antioxidant effects of micronutrients and herbal medicine extractions on both testosterone production and oxidative stress are listed in Supplementary Tables S1 and S2, respectively. Testosterone and oxidative stress reportedly improved in *in vitro* models of Leydig cells treated with different antioxidant compounds [159,160,161,162,163,164,165,166,167,168,169,170]. Similar results were observed across various animal models of induced hypogonadism by using vitamins A and C [171], zinc [172], *N*-acetylcysteine (NAC) [173,174], gallic acid [175], lycopene [176], forskolin [177], resveratrol [167,178], *Nigella sativa* seed oil [179], *Lycium chinense* Mill [180], *Eruca sativa* seeds [181], *Moringa oleifera* leaves [182], *Schisandra chinensis* [183], Ojayeonjonghwan [184,185], and Qilin pills [186] (Supplementary Table S2).

Senolytics are a class of drugs that selectively remove apoptosis-resistant senescent cells through the activation of these apoptosis pathways [187,188]. Naturally derived senolytics include olive phenols, green tea catechins, quercetin, curcumin, and resveratrol [189,190]. Although some of these products have been shown to improve oxidative stress and steroidogenesis in vivo and in vitro (Supplementary Tables S1 and S2), these studies do not selectively report on senescent Leydig cells. However, a synthetic FOXO4-inhibitor as a senolytic agent selectively induced apoptosis in hydrogen-peroxide-induced senescent TM3 Leydig cells and in naturally aged mice [191].

Medicinal plants (phytotherapy) provide secondary metabolites (i.e., flavonoids, polyphenols, catechins, and stilbenes) that are beneficial in the prevention and management of human disease [192,193]. The regular consumption of diets rich in phytonutrients reduces obesity and the related comorbidities, as well as mortality [194]. Various plant secondary metabolites improve oxidative stress in diseases mediated by ROS through direct ROS scavenging, increasing the endogenous antioxidant defences, and through the activation of NF-kβ. Prominent examples include curcumin, garlic extractions, green tea extractions, quercetin, and resveratrol [193]. In diabetes, antioxidants from plants have been found to be beneficial in the inhibition of cellular oxidative stress, with more than 80 plants identified with antidiabetic activity [195]. However, these may also interfere with ROS signalling in glucose metabolism [157,196].

Numerous plants with antioxidant properties have been associated with increased testosterone in males, including improvements in male sexual behaviour, libido, and sexual function. The more common plants investigated include: *Anacyclus pyrethrum*, *Arctium lappa*, *Cinnamomum zeylanicum*, *Coleus forskohlii*, *Corchorus depressus*, *Cynanchum wilfordii*, *Cyperus esculentus*, *Eurycoma longifolia*, *Garcinia cambogia*, *Lepidium meyenii*, *Moringa oleifera*, *Mucuna pruriens*, *Nigella sativa*, *Panax ginseng*, *Pedalium murex*, *Rhodiola rosea* and *Tribulus terrestris*, *Trigonella foenum-graecum Urtica doica*, and *Withania somnifera* [197,198,199,200]. *Trigonella foenum-graecum* and *Withania somnifera* showed promising positive effects in clinical studies, whereas *Lepidium meyenii*, *Rhodiola rosea*, *Tribulus terrestris*, and *Panax ginseng* appeared to have less clinical evidence for increasing male testosterone, but may improve erectile dysfunction [199,200]. More specifically, flavonoids and isoflavonoids may be beneficial in late-onset hypogonadism and the related degenerative diseases associated with ageing. Flavonoids are structured with a molecular backbone, 5,7-dihydroxychromen-4-one, which upregulate StAR expression and activity in the initiation of steroidogenesis [201]. Important flavones include apigenin, chrysin, and luteolin (thyme, celery, and parsley), isoflavones include genistein and daidzein (soybeans), flavonols include quercetin, myricetin, and kaempferol (apples, broccoli, tea, and berries), and naringenin and hesperidin (citrus fruits) [201].

Although oxidative stress is an important mediator in ageing and NCDs, the use of antioxidants in clinical trials have been generally disappointing [7,45,202]. This modest response to antioxidants is termed the "antioxidant paradox", which may be due to a poor understanding of antioxidants in physiology and pathophysiology-related redox biology [202]. Furthermore, antioxidants may exert pro-oxidant activity under certain circumstances, where dosage and cellular redox status are important considerations [203]. An overuse of antioxidants may result in a redox imbalance termed "reductive stress", defined as a decreased ratio of ROS:TAC [204]. Reductive stress can be as damaging as oxidative stress through the induction of mitochondrial dysfunction and DNA damage [205,206]. Hence, the use of antioxidants should be done with appropriate indications, dosages, durations, and monitoring outcomes through empirical evidence [204].

## 6. Conclusions and Future Perspectives

Oxidative stress contributes significantly to the ageing process and the pathogenesis of age- and lifestyle-related NCDs through numerous mechanisms, and is closely related to comorbidities, such as hypertension, dyslipidaemia, hyperglycemia, hyperinsulinemia, and chronic inflammation. Furthermore, evidence suggests that oxidative stress may be a mediator of hypogonadism in these males through the regulation of enzymatic and transcriptional activities. The relationship between ageing, oxidative stress, male hypogonadism, and NCDs in males requires further investigation. These are complex mechanisms that appear to be closely interrelated; hence, a detailed understanding of the impact of ROS on steroidogenesis is required to identify novel therapeutic targets. On the basis of the current evidence, molecular mediators that activate the PI3K/Akt, BMI1, and MAPK family members, as well as p38, may improve Leydig cell steroidogenesis under oxidative stress conditions using nutritional and phytomedicinal antioxidants that require further investigation. In fact, although TRT may be beneficial in these males, alternative therapeutic approaches require consideration where TRT is contraindicated. Weight loss, through nutritional and lifestyle intervention, as well as the use of nutritional and phytomedicinal antioxidants, may provide novel therapeutic options in the management of age-related NCDs in males through an improvement in oxidative stress and steroidogenesis.

## Figures and Tables

**Table 1:** Enzymatic and nonenzymatic antioxidants.

Enzymatic	Nonenzymatic
Superoxide dismutase (SOD)	Vitamin C, vitamin E, vitamin B9
Catalase (CAT)	Selenium, Zinc, Mn^2+^
Glutathione peroxidase (GPx)	Carotenoids, flavonoids, lycopene
Glutathione reductase (GR)	Taurine, hypotaurine
Glutathione-S-transferase (GST)	Glutathione, inositol, cysteine, coenzyme Q10
Thioredoxin	

**Table 2:** A summary comparison of testosterone-replacement therapy (TRT), metformin, caloric restriction, and bariatric surgery impact on obesity, metabolic syndrome, hypogonadism, and oxidative stress.

Management Strategy	Obesity	Metabolic Syndrome	Diabetes	Hypo-gonadism	Oxidative Stress
TRT	Improves [86,87]	Improves [88,89]	Improves [88,89]	Improves [85]	Unclear
Metformin	Improves [102,103]	Improves [102,103]	Improves [102,103]	Unclear	Improves [102,103]
Caloric restriction	Improves [74,132,133]	Improves [74,132,133]	Improves [74,132,133]	Unclear	Improves [129,130]
Bariatric surgery	Improves [138,139,140,141]	Improves [138,139,140,141]	Improves [138,139,140,141]	Improves [138]	Improves [146,147]

## Supplementary Materials

The following are available online at https://www.mdpi.com/article/10.3390/antiox10111834/s1: Table S1: in vitro models investigating antioxidants, micronutrients, or phytonutrients, including both testosterone and oxidative stress outcomes; Table S2: Animal models investigating antioxidants, micronutrients, or phytonutrients, including both testosterone and oxidative stress outcomes.
