# Supplementary material for: The Mechanisms and Management of Age-Related Oxidative Stress in Male Hypogonadism Associated with Non-communicable Chronic Disease
Source: Antioxidants (Basel). 2021 Nov 18;10(11):1834. doi: 10.3390/antiox10111834 (PMC8615233; doi:10.3390/antiox10111834)
Supplement: Supplementary file 1 [file antioxidants-10-01834-s001.zip › antioxidants-1442150-supplementary.pdf]

Supplementary Table S1: In vitro models investigating antioxidants, micronutrients or phytonutrients including both testosterone and oxidative stress outcomes

Antioxidant	Animal Model	Reported Outcomes	Reference
Cerium oxide nanoparticles	Radiofrequency radiation induced oxidative stress in primary mouse Leydig cells	• ↑ testosterone<br>• ↑ StAR and 3β-HSD<br>• ↑ TAC<br>• ↑ CAT<br>• ↓ MDA	[159]
Taurine	di(2-ethylhexyl)phthalate induced oxidative stress and apoptosis in TM3 Leydig cells	• ↑ cellular viability<br>• ↑ testosterone<br>• ↓ MDA<br>• ↓Bax/Bcl2 ratio<br>• ↓ caspase-3 and -9	[160]
Astaxanthin	Hydrogen peroxide induced oxidative stress in TM3 Leydig cells	• ↑ testosterone and progesterone<br>• ↓ intracellular ROS<br>• ↑ StAR expression	[161]
Melatonin	LPS induced inflammation in primary sheep Leydig cells and macrophages co-culture	• ↑ testosterone in Leydig cells<br>• ↑ StAR, 3β-HSD and SF-1 in Leydig cells<br>• ↓ TNF-α, IL-1β, and IL-6<br>• ↑ IL-10<br>• ↓ macrophages secreting inflammatory cytokines and ↑ phagocytic macrophages<br>• ↓ p38/MAPK in macrophages<br>• ↓ TLR4-mediated inflammatory genes in macrophages<br>• ↓ iNOS and NADPH in macrophages	[162]
Resveratrol	Hydrogen peroxide induced oxidative stress in TM3 Leydig cells	• ↑ testosterone and progesterone<br>• ↑ metabolic activity and cell membrane integrity<br>• ↓ intracellular ROS	[163]
	Primary Leydig cells of energy restricted sheep	• ↑ testosterone<br>• ↑ cell proliferation<br>• ↑ steroidogenesis enzymes	[164]
	Benzo(a)Pyrene induced steroidogenic dysfunction in TM3 Leydig cells	• ↑ cellular viability<br>• ↑ testosterone<br>• ↑ expression of StAR, CYP11A1, 3β-HSD and 17β-HSD<br>• ↑ p38 MAPK<br>• ↑ SF1<br>• ↑ SOD1, SOD2, GPx and CAT	[165]
Rutin	Hydrogen peroxide induced oxidative stress in TM3 Leydig cells	• ↑ cell survival rate<br>• ↑ testosterone<br>• ↓ intracellular ROS and LPO<br>• ↓ GPx	[166]

		• ↑ CAT, SOD, peroxidase<br>• ↑ Bcl-2, PI3K and p-AKT<br>• ↓ Bax and caspase-3	
Quercetin	Triptolide induced oxidative stress in rat Leydig cells	• ↑ testosterone<br>• ↓ intracellular ROS<br>• ↑ GPx and SOD<br>• ↓ Apoptosis<br>• ↑ MMP<br>• ↓ BAX/Bcl-2 ratio<br>• ↓ caspase-3 and -9	[167]
Morinda officinalis	Hydrogen peroxide induced oxidative stress in TM3 Leydig cells	• ↑ cellular viability<br>• ↑ testosterone<br>• ↓ LPO<br>• ↑ SOD	[168]
Moringa oleifera	TM3 Leydig cells	• No change in cell viability<br>• ↑ testosterone<br>• ↑ GSH<br>• No change for CAT, SOD and TAC<br>• No change for lipid peroxidation	[169]
Salvia officinalis	TM3 Leydig cells	• ↑ testosterone and progesterone<br>• No induction of oxidative stress except at highest concentrations exposed	[170]

Abbreviations: 3β-HSD: 3β-Hydroxysteroid dehydrogenase; 17β-HSD: 17β-Hydroxysteroid dehydrogenase; Akt: Protein kinase B; Bax: Bcl-2-associated X protein; CAT: catalase; CYP11A1: cytochrome P450 family 11 subfamily A member 1; GPx: glutathione peroxidase; GSH: glutathione; IL: interleukin; iNOS: inducible nitric oxide synthase; LPO: lipid peroxidation; MDA: malondialdehyde; MMP: mitochondrial membrane potential; PI3K: phosphatidylinositol 3-kinases; ROS: reactive oxygen species; SF1: splicing factor 1; SOD: superoxide disumutase; TAC: total antioxidant capacity; StAR: steroidogenic acute regulatory; TLR4: toll-like receptor 4; TNFα: tumor necrosis factor alpha.

Supplementary Table S2: Animal models investigating antioxidants, micronutrients or phytonutrients including both testosterone and oxidative stress outcomes

Antioxidant	Animal Model	Reported Outcomes	Reference
Vitamin A and Vitamin C Combination	Aroclor induced testicular toxicity in adult rats	• ↑ serum testosterone, LH and FSH<br>• ↓ serum prolactin and oestrogen<br>• ↑ LH receptor expression on Leydig cells<br>• ↑ P450scc, 3β-HSD and 17β-HSD in Leydig cells<br>• ↑ SOD, CAT, GPx, GR, γ-GT and GST in Leydig cells<br>• ↓ ROS and LPO in Leydig cells	[171]
Zinc	Cadmium induced testicular toxicity in adult rats	• ↑ serum testosterone and LH<br>• ↑ sperm concentration and motility<br>• ↓ testicular MDA<br>• ↓ testicular TNFα<br>• ↓ testicular NO and iNOS<br>• Improvement in testicular histological damage	[172]
N-acetylcysteine (NAC)	Aroclor induced testicular toxicity in adult rats	• ↑ serum testosterone<br>• ↑ Leydig cell intracellular cAMP<br>• ↓ Leydig cell LPO<br>• ↓ Leydig cell apoptosis<br>• ↓ Leydig cell caspase-3, -8 and -9, Bax, Fas, FasL and JNK/pJNK<br>• ↑ Bcl-2, pAkt/Akt and NF-kβ	[173]
	BMI1 null mice	• ↑ steroidogenesis<br>• ↓ DNA damage<br>• ↓ cellular senescence	[174]
Gallic acid	Cisplatin induced reproductive toxicity in adult rats	• ↑ serum testosterone<br>• ↑ spermatogonia, Leydig and Sertoli cell numbers<br>• ↑ testicular volume<br>• ↑ testicular CAT, SOD and GSH enzymes	[175]
Lycopene	Polychlorinated biphenyl induced testicular toxicity in adult rats	• ↑ serum testosterone<br>• ↑ Leydig cell StAR, P450scc and 3β-HSD expression	[176]
Forskolin	Mancozeb-induced reproductive toxicity in adult rats	• ↑ serum testosterone<br>• ↑ spermatogenesis<br>• ↑ epididymal sperm count<br>• ↑ testicular GSH, SOD and CAT activity	[177]

Resveratrol	Diet induced obesity associated secondary hypogonadism in mice	• ↑ serum testosterone and LH<br>• ↑ testicular StAR and steroidogenic enzyme<br>• ↑ testicular SOD and GPx	[178]
	Benzo(a)Pyrene steroidogenic dysfunction in adult rats	• ↑ serum and intratesticular testosterone<br>• ↓ intratesticular ROS<br>• ↑ Leydig cell expression of StAR, CYP11A1, 3β-HSD and 17β-HSD<br>• ↑ Leydig cell SF1 expression<br>• ↓ Leydig cell DAX production	[165]
Nigella sativa seed oil	Chlorpyrifo-induced reproductive toxicity in adult rats	• ↑ serum testosterone<br>• ↑ semen parameters<br>• ↑ serum GSH and antioxidant enzymes<br>• ↓ serum ROS	[179]
Lycium chinense Mill	Aged rat model of late-onset hypogonadism	• ↑ serum testosterone<br>• ↓ testicular oxidative stress	[180]
Eruca sativa seeds	Silver nanoparticles induced testicular toxicity in adult rats	• ↑ serum testosterone, LH, FSH and prolactin<br>• ↑ testicular GSH, GST, SOD<br>• ↓ testicular serum TBARS<br>• ↓ Leydig cell TNFα concentrations<br>• ↓ testicular DNA fragmentation	[181]
Moringa oleifera leaves	Tramadol induced testicular toxicity in adult rats	• ↑ serum testosterone, LH and FSH<br>• ↑ body weight and testes weight<br>• ↑ sperm counts, vitality, total sperm motility and normal morphology<br>• ↑ testicular CAT and SOD<br>• ↓ testicular TBARS<br>• ↑ spermatogenic cell and Leydig cell numbers	[182]
Schisandra chinensis	Varicocele induced testicular dysfunction	• ↑ serum testosterone<br>• ↓ serum LH and FSH<br>• ↑ testicular weight, sperm count and sperm motility<br>• ↑ testicular Johnsen score and spermatogenic cell density<br>• ↑ testicular SOD, GPx and CAT<br>• ↓ testicular MDA, ROS, RNS<br>• ↓ apoptotic index<br>• ↑ testicular StAR<br>• ↓ Grp 78, p-JNK, p-IRE1α<br>• ↓ cleaved caspase 3<br>• ↓ Bax:Bcl2	[183]
Ojayeonjonghwan (KH-204 - Korean herbal formula)	Aged rat model of late-onset hypogonadism	• ↑ serum testosterone<br>• ↑ serum SOD<br>• ↓ serum 8-OHdG	[184]

	Leuprorelin induced androgen deprived rats	• ↑ serum testosterone<br>• ↑ serum SOD<br>• ↓ serum 8-OHdG<br>• ↑ Leydig cell expression of Nrf2/ HO-1<br>• ↓ expression of TGF-β 1/SMAD	[185]
Qilin pills (Traditional Chinese Medicine herbal formula)	Tripterygium glycoside induced oligoasthenospermia in adult rats	• ↑ serum testosterone, LH, FSH, SHBG<br>• ↑ semen parameters<br>• ↑ testicular SOD<br>• ↓ testicular ROS and MDA<br>• Improved testicular histology	[186]

Abbreviations: 3β-HSD: 3β-Hydroxysteroid dehydrogenase; 8-OHdG: 8-hydroxy-20-deoxyguanosine; 17β-HSD: 17β-Hydroxysteroid dehydrogenase; Akt: Protein kinase B; Bax: Bcl-2-associated X protein; cAMP: cyclic adenosine monophosphate; CAT: catalase; CYP11A1: cytochrome P450 family 11 subfamily A member 1; FasL: Fas ligand; FSH: Follicle-stimulating hormone; GPx: glutathione peroxidase; GR: glutathione reductase; Grp 78: glucose-regulated protein-78; GSH: glutathione; GST: glutathione S-transferase; HO-1: heme oxygenase-1; iNOS: inducible nitric oxide synthase; JNK: c-Jun N-terminal kinase; LH: luteinizing hormone; LPO: lipid peroxidation; MDA: malondialdehyde; NF-kβ: nuclear factor kB; NO: nitric oxide; Nrf2: nuclear factor erythroid 2-related factor 2; P450scc: Cholesterol side-chain cleavage enzyme; p-IRE1α: phosphorylated inositol-requiring transmembrane kinase/endoribonuclease 1α; RNS: reactive nitrogen species; ROS: reactive oxygen species; SF1: splicing factor 1; SHBG: sex hormone binding globulin; SMAD: Mothers against decapentaplegic; SOD: superoxide disumutase; StAR: steroidogenic acute regulatory; TBARS: thiobarbituric acid reactive substances; TGF- β - transforming growth factor-beta; TNFα: tumor necrosis factor alpha; γ-GT: gamma glutamyl transferase.
